# Endoscopic recanalization of complete fibrotic colorectal anastomosis using an endoscopic ultrasound-guided hybrid approach

**DOI:** 10.1055/a-2216-1042

**Published:** 2024-01-17

**Authors:** Jianbo Ni, Linlin Zhang, Yuqing Mao, Shengzheng Luo, Xiaoyuan Gong, Baiwen Li

**Affiliations:** 1 Department of Gastroenterology, Shanghai General Hospital, Shanghai Jiao Tong University School of Medicine, Shanghai, China; 2 56694 Shanghai Key Laboratory of Pancreatic Diseases, Shanghai General Hospital, Shanghai Jiao Tong University School of Medicine, Shanghai, China

Complete benign anastomotic obstructions post-colorectal surgery traditionally demand surgical intervention 1 2 . We report the successful recanalization of a completely fibrotic rectal anastomosis using an unprecedented endoscopic ultrasound (EUS)-guided hybrid approach, which offers a less invasive alternative to surgery.

A 72-year-old man was referred for treatment of complete benign colorectal obstruction. He had previously undergone laparoscopy-assisted low anterior resection with a protective loop ileostomy for treatment of rectal cancer. A fully obstructed anastomosis with surgical staples was revealed by colonoscopy, 4 cm from the anal verge ( Fig. 1 ). After a multidisciplinary discussion, we decided to recanalize the lumen by EUS guidance instead of surgical reintervention.

**Fig. 1:**
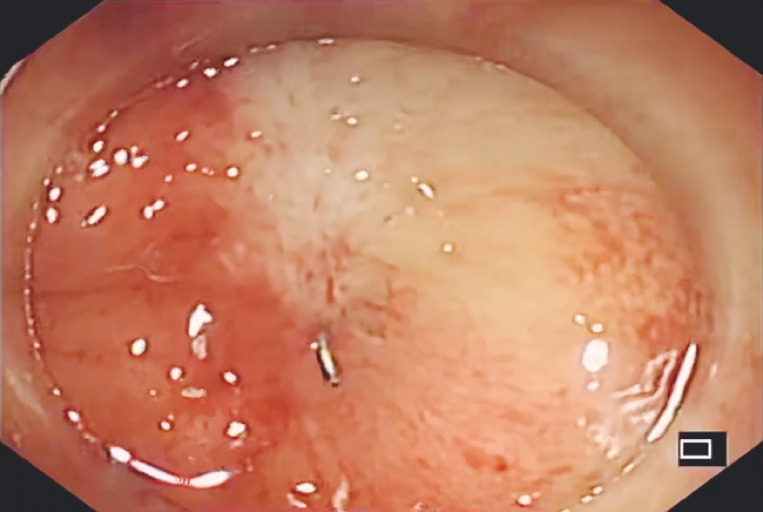
Colonoscopy revealed a complete anastomotic obstruction with surgical staples very close to the anal verge.

The initial EUS-guided rendezvous approach failed because the proximal end of the sigmoid colon could not be accessed via trans-stomal colonoscopy. Thus, direct EUS-guided retrograde puncture was performed.

A therapeutic linear echoendoscope (EU-ME2; Olympus, Tokyo, Japan) was positioned at the end of the anastomosis, and the proximal colon was punctured with a 19-gauge needle (Boston Scientific Corp., Marlborough, Massachusetts, USA) under EUS guidance. The colonic lumen was confirmed by contrast filling on fluoroscopy; sterile normal saline was then infused to extend the lumen. A 0.035-inch guidewire (Boston Scientific Corp.) was advanced to maintain wire access across the lumen and the EUS needle was retrieved. Unfortunately, endoscopic balloon dilation failed owing to severe fibrosis of the anastomosis, coupled with anastomotic staples around the puncture site ( Fig. 2 ). The center of the fibrotic anastomotic membrane could not be perforated by a colonoscope needle knife (MTW Endoskopie, Wesel, Germany).

**Fig. 2:**
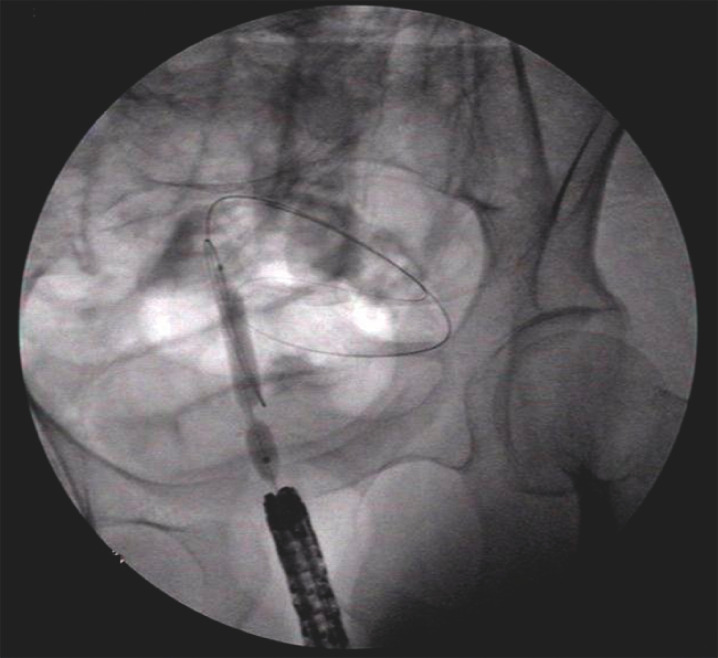
Failure of endoscopic balloon dilation was attributed to severe anastomotic fibrosis and presence of staples near the puncture site.

The endoscopic incision of the fibrotic tissue was meticulously executed using a 1.5-mm DualKnife (Olympus), guided by the direction of the guidewire. The most crucial step involved carefully peeling off the fibrotic tissue surrounding the anastomotic staples and then removing the staples one by one using an endoscopic grasper ( Fig. 3 , Fig. 4 ). The anastomotic stricture was sequentially dilated to a maximum diameter of 10 mm under endoscopic and fluoroscopic guidance. Luminal continuity was re-established, allowing the slim colonoscope to successfully pass through the fistula. To avoid the risks of fluid leaks, stent migration, and fistula re-stenosis, a diabolo-shaped lumen-apposing metal stent (Hot AXIOS; Boston Scientific Corp.), measuring 15×10 mm, was deployed across the colorectal fistula and left in place for 4 weeks ( Fig. 5 ). No immediate or delayed complications were observed.

**Fig. 3:**
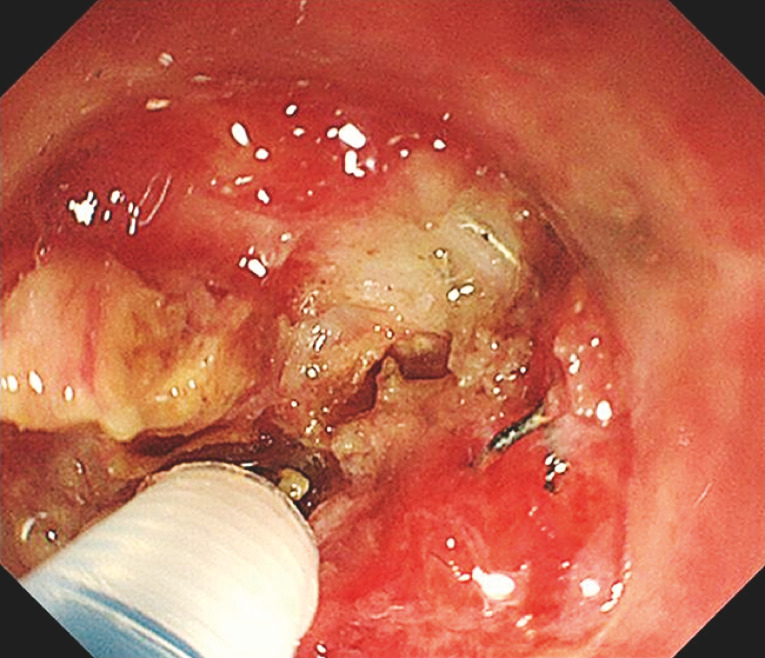
Endoscopic incision of fibrotic tissue was directed by the guidewire.

**Fig. 4:**
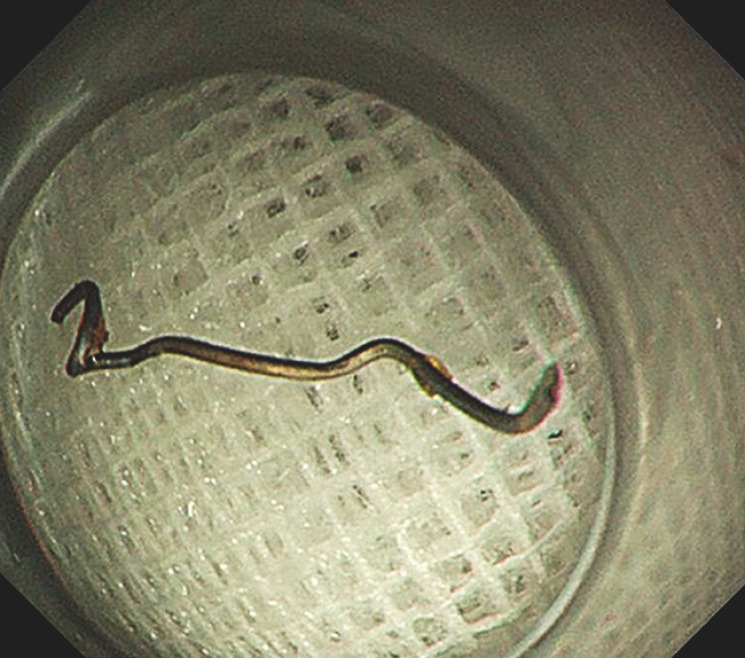
A successfully removed anastomotic staple.

**Fig. 5:**
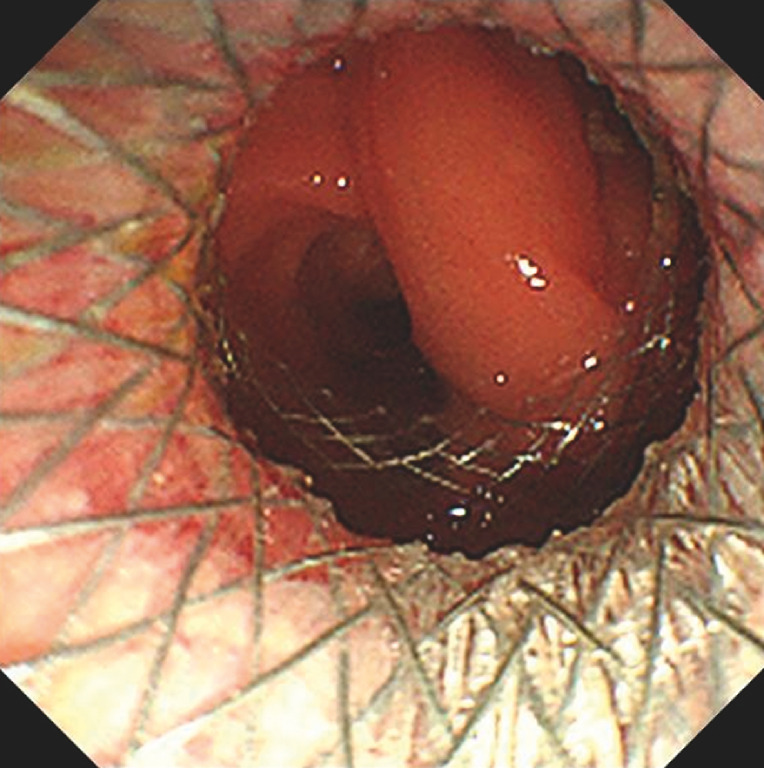
Endoscopic image showing the successfully deployed lumen-apposing metal stent.

The stent was removed by the referring gastroenterologist, followed by ileostomy reversal 2 months after recanalization ( Video 1 ). Clinically, the patient has shown no recurrence of the stenosis at 6-month follow-up intervals.

Endoscopic recanalization of complete fibrotic colorectal anastomosis using an endoscopic ultrasound-guided hybrid approach.Video 1

Complete fibrotic gastrointestinal obstructions are rare and present significant challenges. Endoscopic treatment could prevent the need for additional surgical intervention and improve the quality of life of patients 3 4 5 . In this case, the meticulous procedural steps, including fibrotic tissue incision and anastomotic staple removal, led to a successful outcome. This case highlights the utility and feasibility of a novel EUS-guided hybrid approach, offering a less invasive alternative to surgical intervention. Careful patient selection and technique are essential to minimize the risk of complications.

Endoscopy_UCTN_Code_TTT_1AQ_2AF

## Notes

Endoscopy E-Videos https://eref.thieme.de/e-videos
*E-Videos* is an open access online section of the journal *Endoscopy* , reporting on interesting cases and new techniques in gastroenterological endoscopy. All papers include a high-quality video and are published with a Creative Commons CC-BY license. Endoscopy E-Videos qualify for HINARI discounts and waivers and eligibility is automatically checked during the submission process. We grant 100% waivers to articles whose corresponding authors are based in Group A countries and 50% waivers to those who are based in Group B countries as classified by Research4Life (see: https://www.research4life.org/access/eligibility/ ). This section has its own submission website at https://mc.manuscriptcentral.com/e-videos .
